# Production status and research advancement on root rot disease of faba bean (*Vicia faba* L.) in China

**DOI:** 10.3389/fpls.2023.1165658

**Published:** 2023-06-02

**Authors:** Haitian Yu, Feng Yang, Chaoqin Hu, Xin Yang, Aiqing Zheng, Yubao Wang, Yongsheng Tang, Yuhua He, Meiyuan Lv

**Affiliations:** ^1^ Institute of Food Crops, Yunnan Academy of Agricultural Science, Kunming, Yunnan, China; ^2^ Department of Agricultural, Food and Nutritional Science, University of Alberta, Edmonton, AB, Canada; ^3^ Qujing Academy of Agricultural Sciences, Qujing, Yunnan, China

**Keywords:** *Vicia faba*, production, root rot, identification, management

## Abstract

China is the largest producer of faba bean with a total harvested area of 8.11×10^5^ ha and a total production of 1.69 ×10^6^ tons (dry beans) in 2020, accounting for 30% of the world production. Faba bean is grown in China for both fresh pods and dry seed. East China cultivates large seed cultivars for food processing and fresh vegetables, while northwestern and southwestern China grow cultivars for dry seeds, with an increased production of fresh green pods. Most of the faba bean is consumed domestically, with limited exports. The absence of unified quality control measures and simple traditional cultivation practices contributes to the lower competitiveness of the faba bean industry in international markets. Recently, new cultivation methods have emerged with improved weed control, as well as better water and drainage management, resulting in higher quality and income for producers. Root rot disease in faba bean is caused by multiple pathogens, including *Fusarium* spp., *Rhizoctonia* spp., and *Pythium* spp. *Fusarium* spp. is the most prevalent species causing root rot in faba bean crops and is responsible for severe yield loss, with different species causing the disease in different regions in China. The yield loss ranges from 5% to 30%, up to 100% in severely infected fields. The management of faba bean root rot disease in China involves a combination of physical, chemical, and bio-control methods, including intercropping with non-host crops, applying rational nitrogen, and treating seeds with chemical or bio-seed treatments. However, the effectiveness of these methods is limited due to the high cost, the broad host range of the pathogens, and potential negative impacts on the environment and non-targeted soil organisms. Intercropping is the most widely utilized and economically friendly control method to date. This review provides an overview of the current status of faba bean production in China, the challenges faced by the industry due to root rot disease, and the progress in identifying and managing this disease. This information is critical for developing integrated management strategies to effectively control root rot in faba bean cultivation and facilitating the high-quality development of the faba bean industry.

## 1 Introduction

Faba bean (*Vicia faba* L.), native to the Mediterranean and Central Asia, is an important legume crop that can be used as food for human consumption and as livestock feed (Crépon et al., 2010). The characteristics of high protein content in seed and straw, the high efficacy of root-rhizobia in nitrogen fixation, the good potential in soil quality improvement (Duchene et al., 2017), and the good adaptation in different habitats (Singh et al., 2013) make it well recognized and widely cultivated in the world.

While the production of primary crops such as wheat, rice, maize, and sugarcane has increased 52% from 2000 to 2021 (9.3 billion tons) (FAOSTAT, 2022), the challenges posed by climate change and loss of quality and area of arable land are limiting crop production (Döös, 2002; Shi et al., 2016; Snowdon et al., 2021). Additionally, global water stress and rising hunger (Alcamo et al., 2007; Oxford Analytica, 2019), with most of the undernourished population living in Asia and Africa, are increasing stress on agricultural production. To meet the food needs of the rapidly growing world population, a 70% increase in food production by 2050 is suggested by the FAO (Food and Agriculture Organization of the United Nations) (Kuttibai et al., 2022). The overuse of chemical fertilizers and pesticides to achieve high yield, although to a lesser extent, also impairs agricultural sustainability and human health (Liu and Wu, 2022). As reported by the FAO, over 200 million tons of fertilizers, with 56% nitrogen, were applied in 2020 and pesticide use has increased by 30% since 2000. These factors emphasize the importance of increasing the cultivation and utilization of legume crops in agriculture, which will diversify the agroecosystem, reduce pest stress, lower nitrogen fertilizer inputs, improve soil quality, and increase the availability of legume protein for nourishing the needy population (Costanzo and Bàrberi, 2014; Duchene et al., 2017; Blesh, 2019; Zhang et al., 2019).

The production of legume crops, including faba bean, is crucial to alleviate the increasing challenges in crop production such as global food stress, land degradation, and overuse of chemicals. However, various abiotic and biotic factors can hamper legume productivity (Varshney et al., 2009). Root rot disease associated with Fusarium wilt is considered as one of the major constraints on legume production (Liebenberg, 2002; Infantino et al., 2006; Singh and Schwartz, 2010) and has been reported to cause severe disease in faba bean (Sillero et al., 2010; Hou et al., 2011; Paul and Gupta, 2021). In faba bean production in China, root rot and wilt disease caused by various fungi including *Fusarium* spp., *Rhizoctonia solani*, and *Pythium debaryanum* are a major challenge, leading to yield losses of 5%–30% and even up to 100% under favorable environmental conditions (Dong et al., 2014a; Zhang et al., 2018). Charcoal rot caused by *Macrophomina phaseolina* has also been recently reported in Yunnan province (Sun et al., 2019; Yu et al., 2021). This review summarizes the status of faba bean production in China and the research advancement on root rot and wilt diseases, providing crucial information for future strategies in the development of the faba bean industry and integrated disease management.

## 2 Status of faba bean production of China

**Figure 1:**
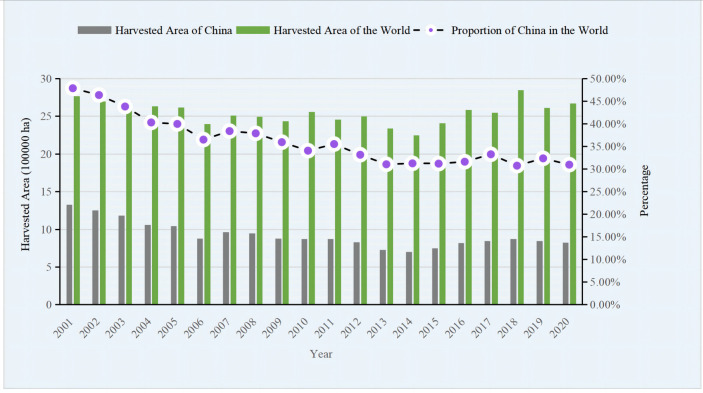
Harvested area of faba bean of china and the World from 2001 to 2020.

**Figure 2:**
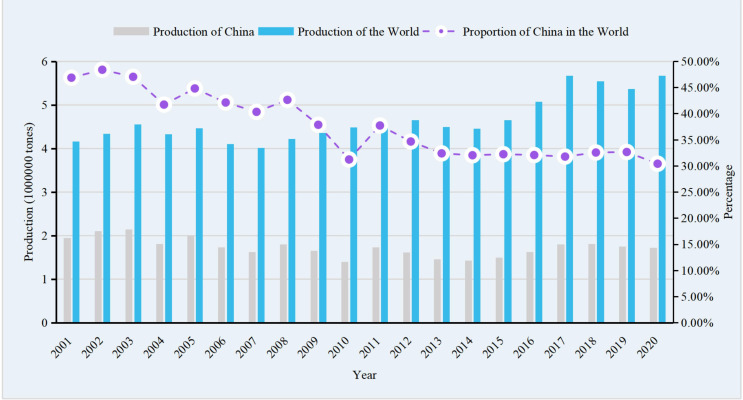
Production of faba bean of China and the World from 2001 to 2020.

**Figure 3:**
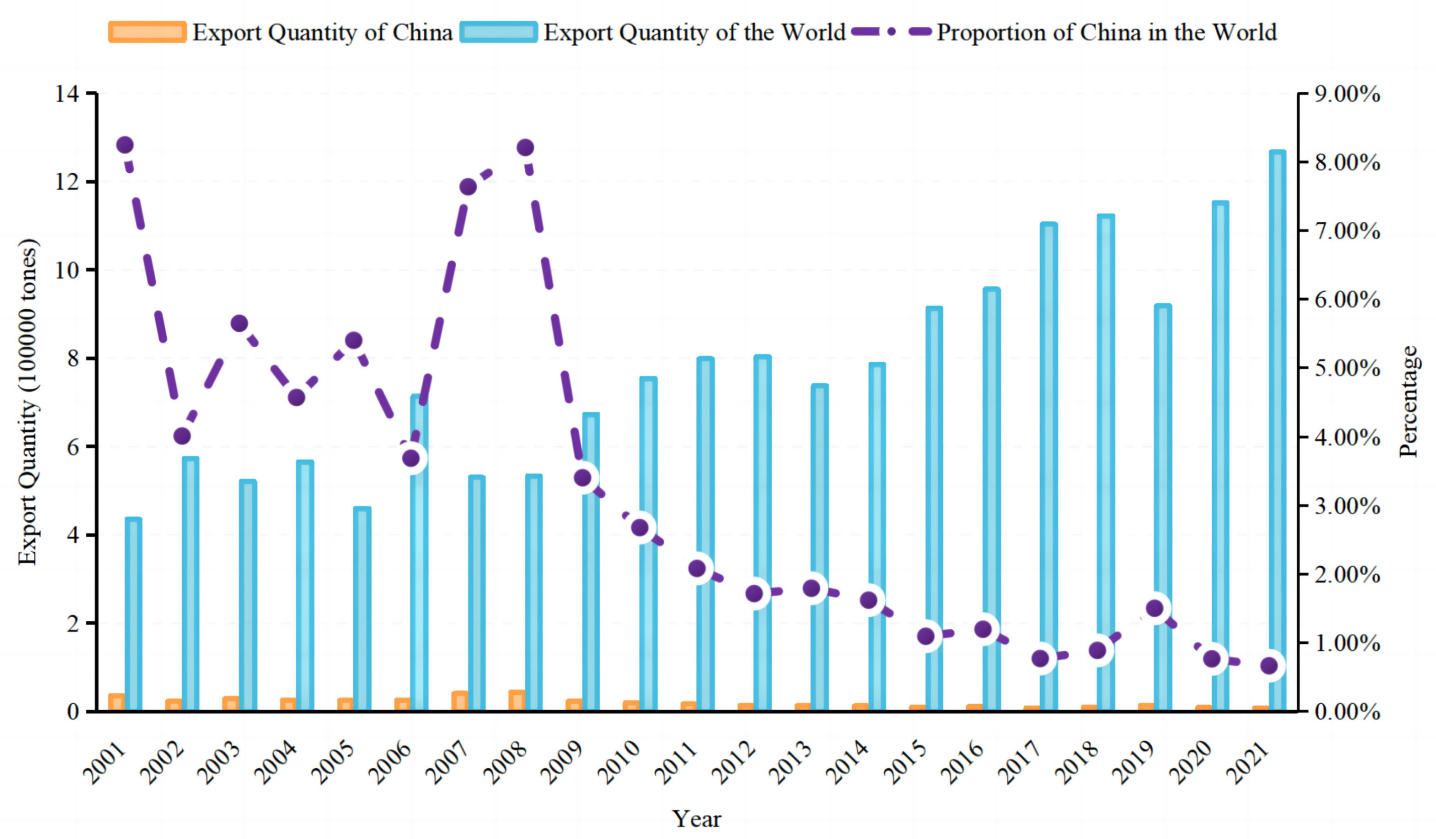
Exporting quantity of faba bean of China and the world from 2001 to 2020.

**Figure 4:**
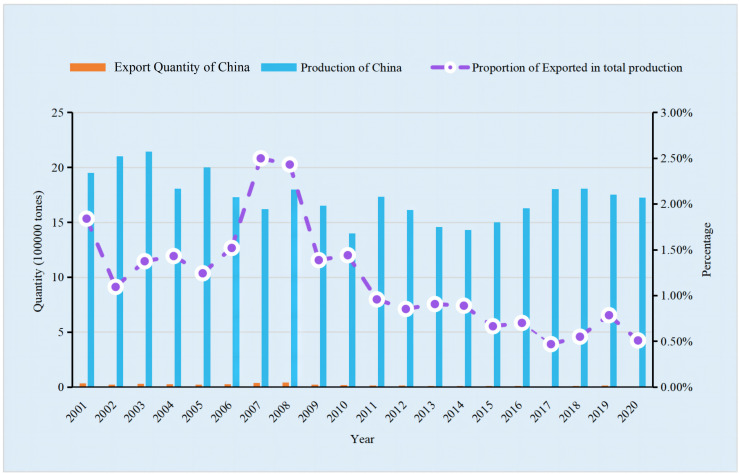
Proportion of Exporting quantity of faba bean in total production of China from 2001 to 2020.

According to data from the FAO from 2001 to 2020, China led the world in faba bean production, with total harvested area and production of dry bean both accounting for more than 30% of the total global amount. In 2020, China and the world produced 1.69×10^6^ and 5.68×10^7^ metric tons of faba beans, respectively, with total harvested areas of 8.11×10^5^ and 2.66×10^6^ ha, respectively (dry beans), followed by Ethiopia, Australia, and the UK (FAOSTAT, 2022). In the past 20 years in China (**Figures 1**, **2**), the lowest area harvested and the resulting production of faba bean was seen in 2014, which then reached a relatively stable level between 2016 and 2020. Compared to 2001, the harvested area and production of faba bean have decreased by 37.62% and 11.61%, respectively, by 2020. The shift in land use in Yunnan province from faba bean cultivation to vegetable cultivation, due to the desire for higher income crops, contributed to the decrease (Yu et al., 2019a). Despite the reduction in the harvested area and production, the increase in average yield of faba bean, from 1.47 tons·ha^−1^ to 2.12 tons·ha^−1^ (FAOSTAT, 2022), has helped compensate for the losses. Furthermore, there is still a huge potential for improvement, as the yield of faba bean in the regional trials in Yunnan was more than 3.0 tons·ha^−1^. In China, most of the fresh beans and over 90% of the dry products were consumed domestically. However, importation of fresh beans has not been reported since 1961 and imports of dry beans, from 2014 to 2020, was limited to a few hundred kilograms, with most of the faba bean imported from ICARDA and used as germplasm for research purposes. Furthermore, the quantity of dry seeds exported out of China accounted for only 0.5%–2.5% of the total production in China and 0.66%–8.24% of the total production in the world (**Figures 3**, **4**). The trend of decreased exports has lasted for more than 10 years (FAOSTAT, 2022). The low level of importation suggests a high degree of self-sufficiency, and a low level of export is attributed to the low consistency and quality of the faba bean product. Conversely, Australia, the third leading producer of faba bean, is the leading exporter (Johnson et al., 2021; Dhull et al., 2022) and is highly competitive in international markets because of consistent, high-quality commodity production (AEGIC, 2019).

Faba bean is traditionally cultivated in most parts of China, except for the northeast provinces of Heilongjiang, Jilin, and Changchun (Ye et al., 2003). The cultivation region of faba bean crosses large latitudinal and longitudinal ranges. The ecotype of faba bean was classified into the winter ecotype (between 21°N and 35°N latitude), basically sown between August and December and harvested from March to May of the next year, and the spring ecotype (between 31°N and 53°N latitude), sown between February and May and maturing in the fall season (Ye et al., 2003; Wang et al., 2012; Yu et al., 2019a). Typically, the northern and northwestern provinces are the regions for the spring ecotype, whereas the central, east, and southwest areas of China normally cultivate the winter faba bean (Lang et al., 1993; Wang et al., 2012). All parts of the faba bean plant, fresh or dry, were well utilized in China, usually eaten as a vegetable (fresh seed, pods, and plant shoots), used as food (dry seed), used in livestock (all parts), and used as a natural nitrogen resource for the agricultural system (all parts) (Ye et al., 2003; Yu et al., 2020), with dry beans being the major product. Cultivation of faba bean in China (**Table 1**) and the intended market is determined by seed size (Ye et al., 2003). Different cultivars have been developed for various purposes, with big seed cultivars [(hundred seed weight (HSW) > 120 g] used for both food processing and fresh vegetable use, while medium (70 g ≤ HSW ≤ 120 g) and small (HSW < 70 g) seed cultivars are mainly used for food processing and as fodder. The traditional landraces have been replaced by newly bred cultivars, which were developed by different agricultural science academies (Bao et al., 2008; Wang et al., 2020a; Xiang et al., 2022; Yu et al., 2019a). Besides the use of traditional cultivars, breeding and testing new germplasm, and breeding cultivars for special use are emerging purposes (Du, 2021; Yu et al., 2019b). For example, in Chongqing, there is a registered cultivar of faba bean that is used both as an ornamental plant, due to its pink color and defined inflorescence, and as a source of dry seed. This makes it unique when compared to other faba bean cultivars that are primarily grown for their pods or seed (Du, 2021).

**Table 1:** Harvested area of faba bean in the main producing province or autonomous region in China.

Province or autonomous region	Harvested area (ha)	Reference
Gansu	6.8×104	Li et al., 2018
Yunnan	>30×104	Yuan et al., 2020
Sichuan	14×104	Xiang et al., 2022
Chongqing	>6.6×104	He, 2019
Jiangsu	13×104	Minor Grain Crops, 2018
Zhejiang	5×104	Minor Grain Crops, 2018
Qinghai	2.6–2.7×104	Zhang et al., 2022

In East China, production of fresh green pods of faba bean has been commercially well-developed (Zhou et al., 2022). The cultivars of Tongcanxian series and Qidou series, the landraces Cixidabaican and Haimendaqingpi, and the introduced cultivar Lingxiyicun are commonly grown in East China, with a harvested area of approximately 7–8×10^4^ ha. In the northwestern part of China, particularly the provinces of Qinghai, Gansu, Ningxia, and Xinjiang, faba bean is typically harvested for dry seed upon reaching maturity. The Qingdou and Lincan series cultivars are commonly grown in this region, with a harvested area of 2.4–3.0 ×10^4^ ha and 7 × 10^4^ ha, respectively. The cultivation area of faba bean in Gansu alone accounts for over 60% of the total spring faba bean cultivation area in China (Li and Nan, 2000; Hou et al., 2011). In the top-producing region of faba bean, located in southwest China, the crop is predominantly grown for dry seed with an increasing area for fresh green pods production (Yu et al., 2019a; Zhou et al., 2022). Yunnan and Sichuan are the first and second largest producers of faba bean in China, respectively, with more than 30% of faba bean cultivated in Yunnan (Yu et al., 2020). In Sichuan, approximately 1.4× 10^5^ ha of faba bean were harvested in 2022 for both fresh and dry pods, with most of the dry faba bean processed into paste (Xiang et al., 2022).

The cultivation of faba bean in these regions usually involves crop rotation with paddy rice, wheat, oil crops, and other spring crops. In some southwest provinces, such as the mountainous region of Yunnan, early autumn and summer season cultivation is becoming more recognized as a special production of fresh faba bean at an altitude of 2,000 m above the sea level (Yu et al., 2019a). Additionally, Yunnan has a distinct advantage over other production regions, as it has a very diverse agricultural ecosystem at altitudes between 1,500 and 3,000 m above sea level that allows for the production of faba bean in multiple seasons, extending the marketing time (Bao, 2016; Yu et al., 2019a). Traditionally, farmers in Yunnan used broadcast sowing with surface soil tillage and crop rotation with dry land crops as well as direct seeding after rice (*Oryza sativa*) harvest. These methods were widely used due to their cost-effectiveness and high efficiency in nitrogen utilization for the succeeding crop. Recently, new cultivation patterns have been developed in Yunnan, including intercropping with perennial fruit trees such as grape, kiwi, date, and berry, as well as mulching-film side seeding, early autumn seeding, and off-season planting methods. These new methods have been recognized for their advantages, such as improved weed control, better capacity in providing or maintaining water and improving drainage, combined with higher quality and income for producers. Compared to traditional methods, these new patterns are more intensive and suitable for commercial and large-scale production of green beans, and have expanded the plantation region to cooler and higher elevation areas (Bao, 2016; Yu et al., 2018; Yu et al., 2019a).

In China, diverse habitats for cultivation provide a strong foundation for the high-quality and high-yield development of the faba bean industry. Over the past two centuries, based on the national developed research system, significant efforts have been made in cultivar breeding and improving cultural practices. However, the cultivation of faba bean in China is still challenged by the varying environmental conditions in different regions. Further research is necessary to enhance cultural practices that are tailored to the specific needs of each region and production purpose. This will contribute to the goal of achieving high-quality and high-yield production of faba bean. In addition, implementing a standardized quality testing system is critical to the growth and development of the faba bean industry in China.

## 3 Identification of root rot disease in China

The root rot disease of faba bean can be caused by a number of different pathogens, including *Fusarium* spp., *Rhizoctonia* spp., *Pythium* spp., *Phoma* spp., and *Aphanomyces* spp. (Rubiales and Khazaei, 2022). The group of pathogens is often referred to as the root rot complex, with *Fusarium* spp. being the most commonly identified species causing foot and root rot, as well as wilt diseases, in faba bean in China (**Table 2**).

**Table 2:** Disease incidence and yield losses caused by *Fusarium* spp. in a major producing province or autonomous region in China.

Province or autonomous region	Disease incidence (%)	Yield loss or plant death rate (%)	Main causal agents	Reference
Gansu	5–15	Up to 50	*Fusarium solani*; *Fusarium avenaceum*; *Fusarium oxysporum*	Hou et al., 2011; Zhang et al., 2018; Zhang et al., 2020; Liu and Wu, 2022
Xinjiang	4–15.5		*Fusarium solani*, *Fusarium incarnatum*, *Fusarium chlamydosporum* var. *fuscum*	Chu et al., 2019; Duan, 2021
Yunnan	20–30		*Fusarium* oxysporum, *Fusarium* avenaceum, *Fusarium* solani	Wang et al., 2002a; Wang et al., 2018
Jiangsu		10–30	*Fusarium oxysporum*, *Fusarium avenaceum*; *Fusarium moniliforme*	Ren et al., 2003
Zhejiang			*Fusarium acuminatum*, *Fusarium oxysporum*, *Fusarium moniliforme*, *Fusarium moniliforme* var. *subglutinans*, and *Fusarium solani*	Bao et al., 1992
Fujian		5–12	*Fusarium* oxysporum, *Fusarium* avenaceum, *Fusarium* moniliforme, and Fusarium equiseti	Wang, 2013
Qinghai	44–68		*Fusarium solani*	Chen, 1999; Wang et al., 2006

In Qinghai province, *Fusarium* spp. was responsible for severe yield loss, which caused wilt disease in the field with disease incidence ranging from 44% to 68% (Chen, 1999). Fusarium wilt, caused by *Fusarium oxysporum*, has been reported, but root rot caused by *Fusarium solani* is considered the major constraint on faba bean production in Qinghai (Wang et al., 2006). In the cold and humid regions of Gansu province, root rot disease caused by *Fusarium* spp. is a major constraint on faba bean production. The disease can result in yield losses of up to 90% under favorable conditions. *Fusarium solani* is the dominant species, followed by *Fusarium semitectum* and *Fusarium dimerum* (Hou et al., 2011). In addition, *Fusarium avenaceum* was also a commonly identified species causing root rot in faba bean crops. Other pathogens, including *Gliocladium roseum*, *F*. *oxysporum*, *Phoma* spp., *Pythium* spp., *Alternaria* spp., and *R*. *solani*, were also identified (Li and Nan, 1996). In Zhejiang province, located in the eastern part of China, the most significant pathogens causing root rot in faba bean were identified as *Fusarium acuminatum*, *F*. *oxysporum*, *Fusarium moniliforme*, *F. moniliforme* var. *subglutinans*, and *F*. *solani*. These species were determined based on their frequency of occurrence and pathogenicity index. Additionally, *F*. *semitectum* and *Fusarium tricinctum* were also confirmed (Bao et al., 1992). In Fujian province, the frequency of stem wilt disease caused by *F*. *oxysporum* increased since 2010, resulting in yield losses of 5% to 12% in 2013, and over 85% of the faba bean fields were infected (Wang, 2013). In Jiangsu province, a large amount of root and stem rot diseases were observed in the field, with 10% to 30% of plants dying and up to 40% in fields with severe infection. *Fusarium* spp. was identified as the major causal agent, the top four isolated species being *F*. *oxysporum*, *F*. *avenaceum*, *F*. *moniliforme*, and *Fusarium equiseti.* All the four species showed high virulence on faba bean (Ren et al., 2003).

**Figure 5:**
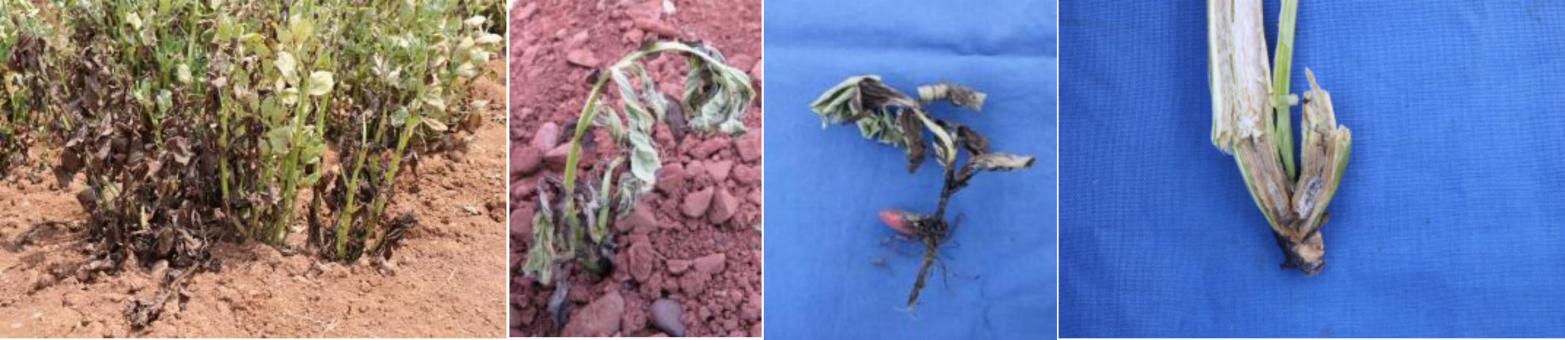
Root rot and wilt symptoms in faba bean in the field of Yunnan.

In Yunnan province, wilt disease, stem rot, and root rot usually occurred in faba bean simultaneously (**Figure 5**), with major pathogens including *F*. *oxysporum*, *F*. *avenaceum*, *F*. *solani*, *R*. *solani*, and *P. debaryanum* Hesee (Wang et al., 2002a). Furthermore, after treatment with the secondary metabolite of *F*. *oxysporum*, the wilting symptom was also clearly observed on pea (*Pisum sativum*), common bean (*Phaseolus vulgaris*), cowpea (*Vigna unguiculata*), and maize (Wang et al., 2002b), which suggested the non-specific toxicity of the pathogen. *Fusarium oxysporum* is dominant at the seedling stage, causing stem rot, while *F*. *avenaceum* is more commonly associated with stem rot and wilt in mature plants (Wang et al., 1988). Based on a provincial survey on faba bean, *Fusarium* spp. is the main genus causing seedling root rot, with *F*. *oxysporum* and *F*. *avenaceum* being the most frequently isolated species. *Rhizoctonia solani* was identified as having the highest virulence among all the isolates, followed by *F*. *oxysporum* and *Fusarium sporotrichioides* (Ruan et al., 1986). Host range studies showed that *F*. *avenaceum*, isolated from the stem of faba bean, caused severe stem and root rot on faba bean and pea, and induced wilt and necrosis symptoms on the leaf of vetch (*Vicia cracce*). However, no infection was found on wheat, maize, common bean, or 35 other crops from 11 different genera (Ruan et al., 1982). Charcoal rot caused by *M. phaseolina* (Tassi) Goid has recently been reported as a problem for faba bean in Yunnan, causing serious yield losses due to root rot, leaf chlorosis, and wilting; eventually, plant death occurred with the necrosis leaf attached (Sun et al., 2019; Yu et al., 2021).

In Xinjiang province, root rot disease on faba bean has been a significant problem since 2009, leading to yield losses of nearly 100% in severely infected fields. The top three prevalent pathogens were identified as *Fusarium* spp., *Rhizoctonia* spp., and *Alternaria* spp. through molecular and morphological characterization (Duan, 2021). Additionally, *Fusarium chlamydosporum* var. *fuscum* was reported to cause severe root and basal stem rot diseases (Chu et al., 2019). In Hubei province, *Fusarium proliferatum* was reported as a causal agent of faba bean root rot (Zhao et al., 2011).

Generally, root rot and wilt diseases are prevalent in faba bean cultivation regions across China, which can cause 100% yield loss under severe conditions. The predominant causative agent of these diseases is the genus *Fusarium*, with different species dominating in different regions. Similarly, *Fusarium* spp. has been reported as the most common pathogen causing foot and root rot in faba bean globally (Sillero et al., 2010; Šišić et al., 2020). However, the characterization of *Fusarium* spp. at diverse taxonomic levels and the impact of the pathogens on the disease development have been investigated to a lesser extent in China and worldwide, although it is crucial for the development of resistant varieties and integrated management strategies. Additionally, most of the studies conducted in China were carried out many years ago, highlighting the need for more recent studies to obtain more accurate information on the causal agents.

## 4 Management of root rot disease in China

Management strategies for root rot have included fungicide treatments, crop rotation, and variety selection, with the most cost-effective strategy being the use of resistant varieties (Marburger et al., 2014; Dolatabadian et al., 2022). However, the availability of resistance to root rot is limited (Rubiales and Khazaei, 2022). Crop rotation can also be an effective strategy but is limited by the broad host range of the pathogens (Bullock, 1992; Cook, 2006; Hwang et al., 2009; Marburger et al., 2014). Fungicide seed treatments are widely used but their efficacy varies depending on the specific species of *Fusarium* and may also have negative impacts on soil organisms and the environment (Munkvold and O’Mara, 2002; Broders et al., 2007; Ellis et al., 2011; Esker and Conley, 2012; Chang et al., 2014).

The management of faba bean root rot disease in China involves various methods, including physical, chemical, and bio-control approaches. One widely used strategy is intercropping with non-host crops, such as wheat, which has been shown to increase the diversity of rhizosphere fungi, reduce the incidence of faba bean root rot disease, and decrease the presence of *Fusarium* spp. in the soil (Luo et al., 2012). Intercropping with wheat has also been reported to decrease the content of citric and malic acid in the rhizosphere, resulting in reduced incidence and severity of Fusarium wilt disease caused by *F*. *oxysporum* (Xiao, 2013). Moreover, intercropping with wheat has been shown to increase the diversity of the rhizosphere microorganism, promote plant tissue integrity and growth, suppress the cinnamic acid-induced stress, alleviate the autotoxicity of faba bean, and increase the gene copy number of *Bacillus brevis*, which can alleviate the effects of Fusarium wilt on the faba bean crop (Dong et al., 2013a; Dong et al., 2017; Lv et al., 2020; Wang et al., 2020b; Guo et al., 2021; Zhang et al., 2023). Furthermore, intercropping faba bean with different wheat cultivars, such as Yunmai 42 and Yunmai 47, has been shown to significantly reduce the disease index of Fusarium wilt and improve rhizosphere microbial activity and diversity. The significant increase in the total content of organic acids and reduction in the levels of soluble sugar and free amino acids in the root exudates of Yunmai 42 and Yunmai 47 were identified as the main reason for the reduction in Fusarium wilt disease (Yang et al., 2014). In field conditions in Gansu, intercropping faba bean with potato in a 2:2 row ratio reduced Fusarium wilt incidence by 5.66% and disease index by 1.6 (Zhang et al., 2020). Faba bean density was also tested, with results showing that 12×10^5^ plants per hectare had the lowest disease incidence and index, and the highest hundred seed weight and yield (Zhang et al., 2018). Under controlled environmental conditions, faba bean grown in soil collected from diseased fields had the lowest plant death rate at 50% water holding capacity (WHC), while growth parameters were significantly better at 50% WHC than at 30% or 70% WHC (Li and Nan, 2000).

The application of nitrogen has been found to be an effective method to control Fusarium wilt in faba bean by altering the composition and metabolic function of the rhizospheric microbial community and reducing the density of *F*. *oxysporum* (Dong et al., 2013b). In Zhejiang, a field study was conducted to investigate the effect of a 3% plant activator protein extracted from *Alternaria* spp. on root rot caused by *F*. *solani*. The results showed that leaf application of a 1,000-times diluted plant activator protein at the seedling stage reduced the disease index by 85.5% compared to the non-treated control (Wu et al., 2006). Similarly, the use of root exudates from different faba bean cultivars can increase resistance to Fusarium wilt by reducing the total content of free amino acids and soluble sugar and increasing organic acids (Dong et al., 2014b).

Inoculation of faba bean with arbuscular mycorrhizal fungi (AMF) species has also been found to enhance the plant’s ability to resist Fusarium wilt and improve microbial carbon metabolic activity in the rhizosphere soil (Dong et al., 2019). Additionally, some rhizobacteria and *Bacillus subtilis* strains have been shown to inhibit the growth of *F*. *oxysporum* and *F*. *chlamydosporum* var. *fuscum*, respectively, which are known to cause root rot in faba bean (Wang et al., 2018; Chu et al., 2019). In Xinjiang, the use of bio-control agents, either as seed treatment, root irrigation, or applied at the time of seeding, showed a significant reduction in disease incidence and an increase in yield (Duan, 2014). The results of a study conducted in Qinghai indicated that the use of two biological pesticides, *Paenibacillus polymyxa* and *Trichoderma harzianum*, was effective in reducing the disease index of Fusarium wilt in faba bean. Application of 1 billion cfu·g^−1^ of *P*. *polymyxa* reduced the disease index by 74.23%, while application of ≥200 million live spores·g^−1^ of *T*. *harzianum* reduced it by 71.01% (Zhang et al., 2022).

A 2-year field study on faba bean root rot control found that applying triadimefon (0.01 g·kg−1 seed) showed the best efficacy, reducing disease index by 51.5% and death rate of mature plants by 31.9%–36%, while increasing seed yield by 19.6%–97.6% (Nan et al., 2002). *In vitro* tests demonstrated that tebuconazole and prochloraz were the most effective fungicides in inhibiting the growth of *Fusarium* spp. and the germination of conidia spores. In field trials, the best seed or root treatment was found to be a combination of prochloraz and *B. subtilis* at a ratio of 1:1 (v:v), with concentrations of 10^4^ μg·ml^−1^ and 2×10^10^ cfu·ml^−1^, respectively. This treatment reduced the disease index by 44.67%–52.21% across several field sites (Ren, 2002).

In China, a few cultivars with moderate to high resistance to *F*. *oxysporum* have been mentioned (Dong et al., 2014b), but the genetic basis of resistance has yet to be explored. Similarly, in Egypt, sources of resistance to *F*. *oxysporum* and *F*. *solani* were reported in faba bean, mostly moderate resistance, but no further studies were conducted to explore the genetic mechanisms (Ali et al., 2019; Mahmoud and Abd El-Fatah, 2020).

In summary, various methods have been found to be effective in reducing the incidence of root rot and wilt disease in faba bean in China. Most of the research has focused on the pathogen *F. oxysporum*, and control of other *Fusarium* species and other pathogens has been less investigated. In Egypt, intercropping faba bean with garlic, as well as with onion and caraway, along with AMF inoculation can reduce root rot disease and enhance profitability and sustainable production (Abdel-Monaim and Abo-Elyousr, 2012; Mousa and El-Sayed, 2016; El-Mehy et al., 2022). Bio-control agents, such as *Paenibacillus* spp.*, Bacillus* spp., and *Trichoderma* spp., all exhibited good potential in suppressing root-related disease in faba bean (Alfauomy and Atwa, 2020). Although biological control has shown promising results on the control of root rot disease, it has not been widely applied in faba bean production because of its cost. Chemical control is widely used, but the timing of application is crucial, as it can be difficult to control the disease once it has already started in the field. While resistance to stem, foot, and root rots has been reported in some germplasms of faba bean, no genetic information was reported. Understanding the genetic mechanism of resistance to *Fusarium* species and pathogens from other genera is essential for the development of effective disease control strategies, including resistance breeding. Developing an integrated management strategy that takes into account multiple factors and adopts a holistic approach is crucial to effectively controlling root rot disease in faba bean in China.

## 5 Conclusion

As a leading country, China plays an irreplaceable role in faba bean production worldwide. The long history of cultivation and diverse habitats across the country allow year-round and multipurpose production to meet domestic needs. However, the lack of standard criteria for quality control, as well as traditional extensive cultural practices, lowers the competitiveness of faba bean products in international markets. Root rot and wilt disease pose another threat to the development of the faba bean industry. Studies on the identification and control of these diseases are still in the primary stage, with little systemic understanding of exploiting the diversity of the causal agents to integrated disease management strategies. To facilitate the faba bean industry development domestically or internationally, it is extremely important to set unified standards for agricultural products, change cultural practices to more intensive methods, and conduct systemic projects to explore knowledge regarding the characteristics of the pathogens involved in root-related diseases and the integrated disease control methods for a specific single pathogen or for a pathogen complex.

## Author contributions

HY: drafting the manuscript. FY, CH, and XY: analysis and/or interpretation of data. AZ, YT, and YW: acquisition of data. AS: revising the manuscript. HY, YH, and ML: revising the manuscript critically for important intellectual content and approval of the version of the manuscript to be published. All authors contributed to the article and approved the submitted version.
